# Camrelizumab Plus Apatinib in Patients With Advanced Cervical Cancer (CLAP): A Multicenter, Open-Label, Single-Arm, Phase II Trial

**DOI:** 10.1200/JCO.20.01920

**Published:** 2020-10-14

**Authors:** Chunyan Lan, Jingxian Shen, Yin Wang, Jundong Li, Zhimin Liu, Mian He, Xinping Cao, Jiayu Ling, Jiaming Huang, Min Zheng, Guorong Zou, Haowen Yan, Qing Liu, Fan Yang, Wei Wei, Yanhong Deng, Ying Xiong, Xin Huang

**Affiliations:** ^1^ Department of Gynecologic Oncology, Sun Yat-sen University Cancer Centre, Guangzhou, China; ^2^ State Key Laboratory of Oncology in South China, Collaborative Innovation Centre for Cancer Medicine, Guangzhou, China; ^3^ Department of Radiology, Sun Yat-sen University Cancer Centre, Guangzhou, China; ^4^ Department of Obstetrics and Gynecology, First Affiliated Hospital, Sun Yat-sen University, Guangzhou, China; ^5^ Department of Radiotherapy, Sun Yat-sen University Cancer Centre, Guangzhou, China; ^6^ Department of Medical Oncology, Sixth Affiliated Hospital, Sun Yat-sen University, Guangzhou, China; ^7^ Department of Radiotherapy, Panyu Central Hospital, Guangzhou, China; ^8^ Department of Cancer Prevention Centre, Sun Yat-sen University Cancer Centre, Guangzhou, China

## Abstract

Camrelizumab is an antibody against programmed death protein 1. We assessed the activity and safety of camrelizumab plus apatinib, a tyrosine kinase inhibitor of vascular endothelial growth factor receptor-2, in patients with advanced cervical cancer.

**METHODS:**

This multicenter, open-label, single-arm, phase II study enrolled patients with advanced cervical cancer who progressed after at least one line of systemic therapy. Patients received camrelizumab 200 mg every 2 weeks and apatinib 250 mg once per day. The primary end point was objective response rate (ORR) assessed by investigators per RECIST version 1.1. Key secondary end points were progression-free survival (PFS), overall survival (OS), duration of response, and safety.

**RESULTS:**

Forty-five patients were enrolled and received treatment. Median age was 51.0 years (range, 33-67 years), and 57.8% of patients had previously received two or more lines of chemotherapy for recurrent or metastatic disease. Ten patients (22.2%) had received bevacizumab. Median follow-up was 11.3 months (range, 1.0-15.5 months). ORR was 55.6% (95% CI, 40.0% to 70.4%), with two complete and 23 partial responses. Median PFS was 8.8 months (95% CI, 5.6 months to not estimable). Median duration of response and median OS were not reached. Treatment-related grade 3 or 4 adverse events (AEs) occurred in 71.1% of patients, and the most common AEs were hypertension (24.4%), anemia (20.0%), and fatigue (15.6%). The most common potential immune-related AEs included grade 1-2 hypothyroidism (22.2%) and reactive cutaneous capillary endothelial proliferation (8.9%).

**CONCLUSION:**

Camrelizumab plus apatinib had promising antitumor activity and manageable toxicities in patients with advanced cervical cancer. Larger randomized controlled trials are warranted to validate our findings.

## INTRODUCTION

Cervical cancer is the fourth-leading cause of cancer-related death in women worldwide.^1^ In China, it was estimated that there were 98,900 new cases of cervical cancer and 30,500 cervical cancer-related deaths in 2015.^2^ The prognosis in women with metastatic or recurrent cervical cancer remains poor,^3,4^ and platinum-based chemotherapy is the first-line treatment. In the GOG 240 trial, the addition of bevacizumab to the first-line treatment significantly improved median overall survival (OS; 17.0 months), compared with chemotherapy (13.3 months, *P* = .004).^4^ In patients who progressed after first-line therapy, bevacizumab,^5^ docetaxel,^6^ topotecan,^7^ and albumin-bound paclitaxel,^8^ have been evaluated; however, the objective response rates (ORRs) were low, and the duration of response was short. Hence, effective therapies for patients with advanced cervical cancer must be developed.

**Context**

**Key Objective**
We aimed to evaluate the antitumor activity and safety profile of a combination therapy using camrelizumab and apatinib as second-line, or later, therapy in patients with advanced cervical cancer. To our knowledge, this is the first study that assessed the combination therapy of an anti–programmed death protein 1 (PD-1) antibody and a vascular endothelial growth factor (VEGF) receptor inhibitor in this setting.
**Knowledge Generated**
The combination of camrelizumab and apatinib showed promising activity, with a favorable response rate and durable response and a manageable toxicity profile in patients with advanced cervical cancer. The activity of this combination was superior to that reported for anti–PD-1/programmed death-ligand 1 antibody or VEGF pathway inhibitor monotherapy alone.
**Relevance**
The promising activity of the combined therapy shown in our study supports the investigation of a camrelizumab plus apatinib combination regimen in a larger randomized controlled trial.

Persistent infection with high-risk human papillomavirus (HPV) is the main cause of cervical cancer. HPV oncoproteins and nonviral tumor antigens have been identified as targets for immunotherapy.^9,10^ In squamous cell carcinoma (SCC), a predominant histologic subtype accounting for approximately 80% of cervical cancer, programmed death-ligand 1 (PD-L1) expression varies from 51% to 88%.^11,12^ These findings provide a rationale supporting the development of immunotherapy in cervical cancer. Recently, the efficacy of immune checkpoint inhibitors has been reported, and pembrolizumab has been approved as a second-line treatment in advanced PD-L1–positive cervical cancer.^13-15^ However, the responses achieved by programmed death protein 1(PD-1) inhibitors were modest.

Angiogenesis is a validated target in the treatment of advanced cervical cancer.^4,5^ Preclinical and clinical studies indicated that antiangiogenic therapy improved the efficacy of immune checkpoint inhibitors.^16^ Apatinib selectively inhibits vascular endothelial growth factor (VEGF) receptor (VEGFR) 2 and showed activity in advanced cervical cancer in retrospective reports.^17-19^ Camrelizumab is a fully humanized, high-affinity monoclonal antibody against PD-1. It possesses clinical activity and a favorable safety profile in cancers.^20,21^ In this phase II study, we assessed the antitumor activity and safety of camrelizumab plus apatinib as second-line, or later, therapy in patients with advanced cervical cancer. This is a proof-of-concept trial with a single-arm, Simon’s two-stage design to detect the preliminary evidence of efficacy and safety profile of this combination therapy.

## METHODS

### Study Design and Participants

The CLAP study is an open-label, single-arm, phase II trial of camrelizumab plus apatinib that was conducted at four academic medical centers in China. The antitumor activity and safety of camrelizumab plus apatinib in patients with advanced cervical cancer were evaluated. The trial protocol was approved by the central and local institutional review boards of all participating centers and the trial was conducted in accordance with the Declaration of Helsinki and Good Clinical Practice guidelines. All patients provided written informed consent before enrolment.

Eligible patients were 18-70 years of age, with histologically confirmed metastatic, recurrent, or persistent cervical cancer that had progressed after at least one line of systemic therapy; had measurable disease according to RECIST version 1.1; and had an Eastern Cooperative Oncology Group performance status score of 0 or 1. Patients also had to have adequate bone marrow, renal, blood coagulation, cardiac, and liver functions. Key exclusion criteria were uncontrolled blood pressure; previous treatment with apatinib; anti–PD-1/PD-L1 or anticytotoxic T-lymphocyte–associated antigen-4 antibodies; active or a history of autoimmune disease; active brain metastases; and active hepatitis B or hepatitis C virus infection.

### Study Treatment

Patients received camrelizumab 200 mg intravenously every 2 weeks and apatinib 250 mg orally once per day continuously in 4-week cycles (maximum of 24 months camrelizumab treatment). The doses were chosen on the basis of a phase I study in advanced cancers.^21^ Treatment continued until disease progression, unacceptable toxicity, or withdrawal of consent. Dose reductions of camrelizumab were not allowed. Dose interruptions and dose reductions (maximum of two reductions) of apatinib were permitted for toxicities that were not relieved by supportive care. The first dose reduction was to 250 mg once per day with 2 days on and 1 day off, and additional reduction was to 250 mg once per day every other day. If the apatinib dose was reduced, it could not be increased later.

### Assessments

Responses were assessed by investigators and radiologists according to RECIST version 1.1 using computed tomography or magnetic resonance imaging at baseline, every two cycles (8 weeks) for the first 10 treatment cycles (40 weeks), and every three cycles (12 weeks) thereafter. Tumor responses had to be confirmed with a repeat scan at least 4 weeks later. Adverse events (AEs) were monitored throughout the treatment period and 30 days after treatment discontinuation (90 days for serious AEs) and were graded according to the National Cancer Institute Common Terminology Criteria for Adverse Events version 4.03.

Tumor PD-L1 expression was assessed using the PD-L1 22C3 pharmDx assay (Dako, Agilent Technologies, Santa Clara, CA) and measured using combined positive score (CPS), defined as the number of PD-L1 staining cells divided by the total number of viable tumor cells, multiplied by 100. PD-L1 positivity was defined as a CPS ≥ 1. HPV status was tested using a p16 mouse monoclonal antibody (CINtec Histology Kit, clone E6H4, Ventana, PA) and was considered positive if more than 90% of the tumor cells showed strong diffuse nuclear and cytoplasmic staining.

### End Points

The primary end point was ORR, defined as the proportion of patients with complete response (CR) or partial response (PR) according to RECIST version 1.1, as assessed by investigators. All responses were confirmed by a second assessment. Secondary end points were progression-free survival (PFS; time from treatment initiation to disease progression according to RECIST version 1.1 or death from any cause), OS (time from treatment initiation to death from any cause), duration of response (time from first evidence of response to disease progression), disease control rate (DCR; the proportion of patients who achieved CR, PR, or stable disease), and safety and tolerability.

### Statistical Analysis

A Simon’s two-stage optimal design was used to test the null hypothesis of a 17% ORR, the historical response rate to pembrolizumab in patients with PD-L1–positive advanced cervical cancer,^13^ against the desired alternative ORR of 35%. This had a one-sided type I error rate of 5% and a power of 80%. In the first stage, 16 patients were accrued. If more than three responders were observed, an additional 28 patients would be accrued to the second stage. The study was considered positive if more than 12 responders were observed among the 44 patients.

Analysis of ORR was performed in both the intention-to-treat (ITT) population, defined as all enrolled patients, and the efficacy-evaluable population, defined as all patients who had received at least one dose of study treatment and had at least one available post-baseline tumor assessment. Safety analyses were performed in all patients who had received at least one dose of study treatment (safety population). ORR and 95% CIs were calculated using the Clopper-Pearson method. The duration of response, PFS, and OS were analyzed using the Kaplan-Meier method. Summary statistics were provided for clinical and demographic characteristics and for AEs. In post hoc analyses, we assessed the association between ORR and exploratory subgroups using the χ^2^ test or Fisher’s exact test and estimated the PFS in exploratory subgroups with the Kaplan-Meier method, and we compared them using log-rank tests. We performed all statistical tests using SAS (version 9.4).

## RESULTS

**FIG 1.**
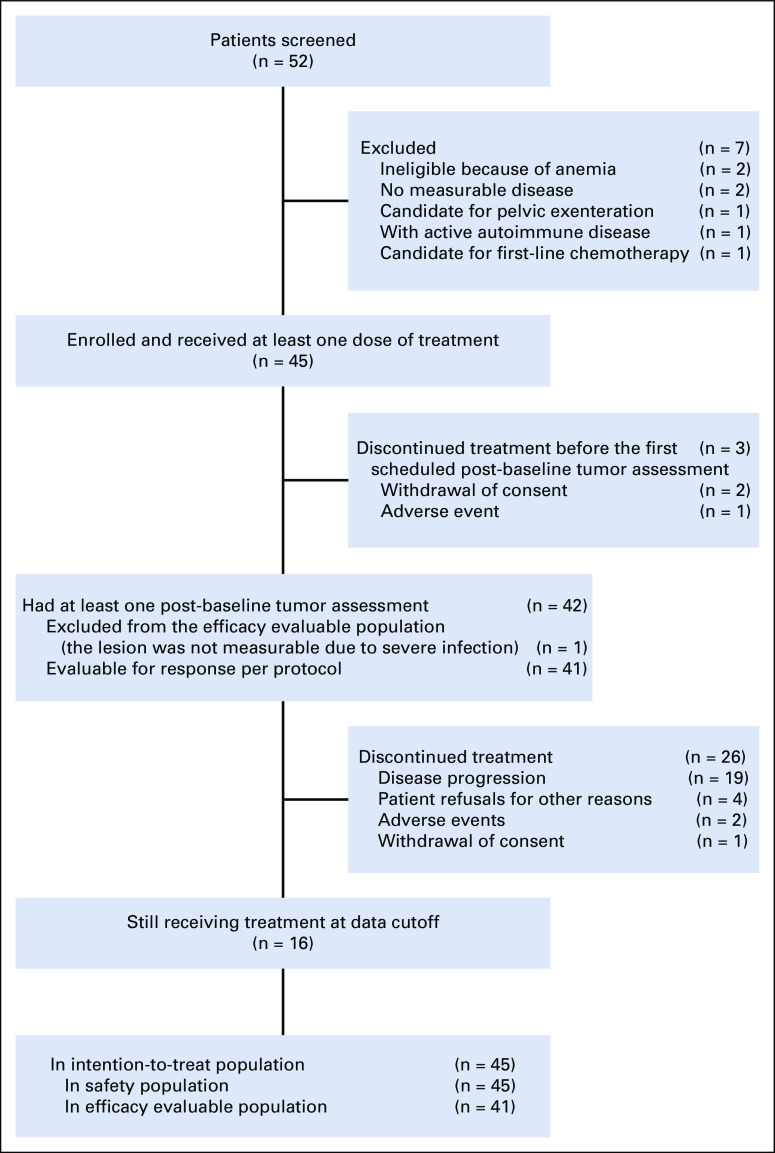
Trial profile.

Between January 21 and August 1, 2019, we screened 52 patients, of whom 45 eligible patients were enrolled and received study treatment (ITT population and safety population). Three patients (6.7%) discontinued treatment before the first scheduled post-baseline scan, and 42 (93.3%) had at least one post-baseline tumor assessment, of which one was not evaluable because of the severe infection of the target lesion. Therefore, 41 patients (91.1%) were included in the efficacy-evaluable population (Fig 1). As of data cutoff (April 30, 2020), the median follow-up was 11.3 months (range, 1.0-15.5 months). Twenty-nine patients (64.4%) discontinued treatment because of disease progression (n = 19 [42.2%]), AEs (n = 3 [6.7%]), withdrawal of consent (n = 3 [6.7%]), and patient refusal (n = 4 [8.9%]). The history of the seven patients who discontinued treatment for reasons other than disease progression and AEs is summarized in the Data Supplement (online only). Baseline characteristics of the population are summarized in Table 1.

**TABLE 1.**
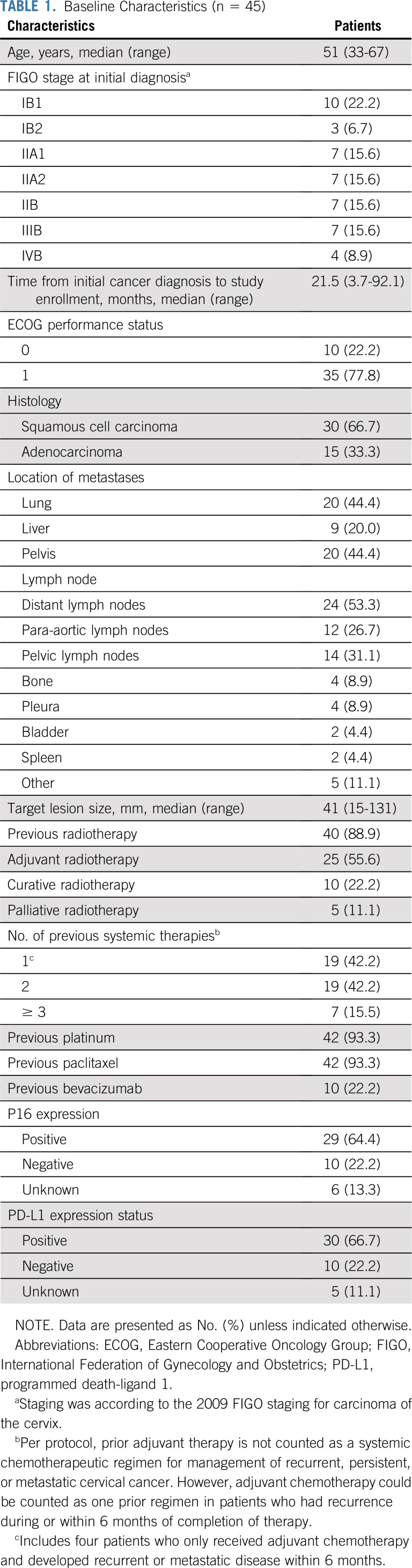
Baseline Characteristics (n = 45)

### Antitumor Activity

**FIG 2.**
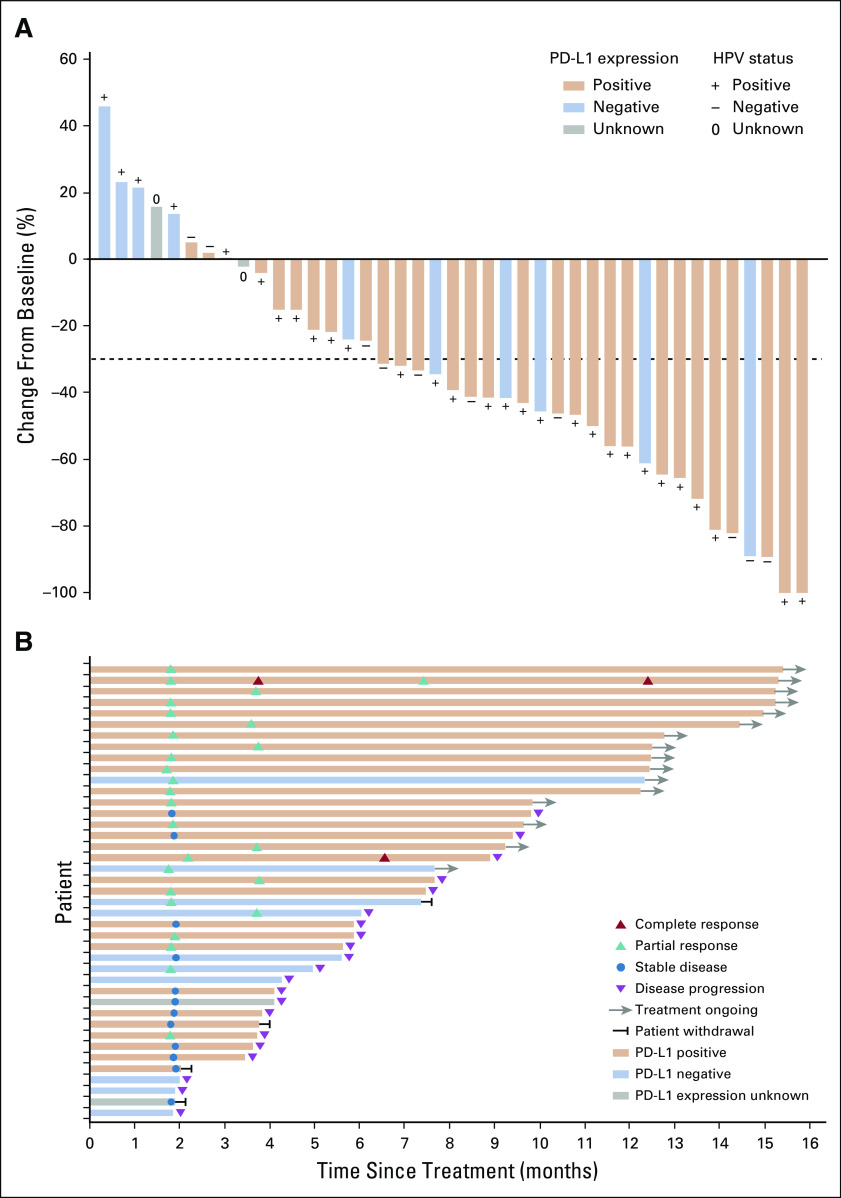
Antitumor activity. The patients in the efficacy evaluable population are included (n = 41). (A) Best percentage change from baseline in target lesion. The dashed line at –30% change represents the RECIST version 1.1 cutoff to define partial response or complete response. (B) Duration of responses. The length of each bar represents the duration treatment of each patient. HPV, human papillomavirus; PD-L1, programmed death-ligand 1.

**FIG 3.**
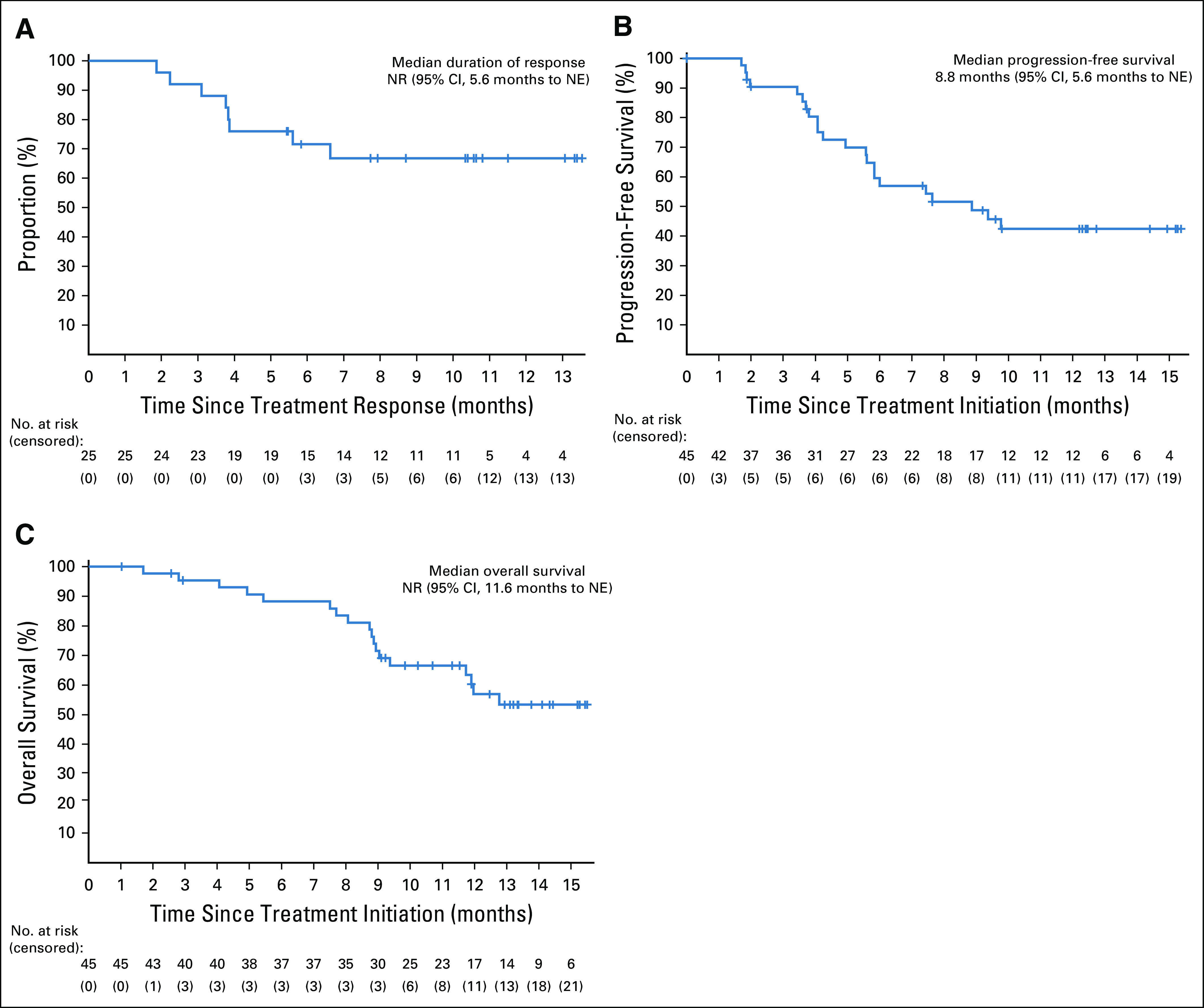
Kaplan-Meier curves of duration of response, progression-free survival, and overall survival. (A) Duration of response was assessed in responders (n = 25), and (B) progression-free survival and (C) overall survival were assessed in the intention-to-treat population (n = 45). NE, not estimable; NR, not reached.

In the first 16 patients enrolled, confirmed responses were noted in eight patients. The ORR threshold for the first stage of Simon’s two-stage was reached, and the trial continued to full accrual. In the ITT population (n = 45), 25 patients (55.6% [95% CI, 40.0% to 70.4%]) achieved a confirmed objective response, with two CRs (4.4%) and 23 PRs (51.1%; Table 2). The DCR was 82.2% (95% CI, 67.9% to 92.0%; Table 2). Similar results were observed in the efficacy-evaluable population (Table 2). In the efficacy-evaluable population, 33 patients (80.5%) had a decrease from baseline in target lesion size (Fig 2A). Among the 25 patients with confirmed objective response, the median time to achieve response was 1.9 months (range, 1.8-3.8 months; Fig 2B). The median duration of response was not reached (95% CI, 5.6 months to not estimable; Fig 3A). Sixteen (64%) of 25 responses were ongoing, with 71.5% of responses (95% CI, 49.3% to 85.3%) lasting at least 6 months and 66.8% of responses (95% CI, 44.2% to 81.9%) lasting at least 12 months.

**TABLE 2.**
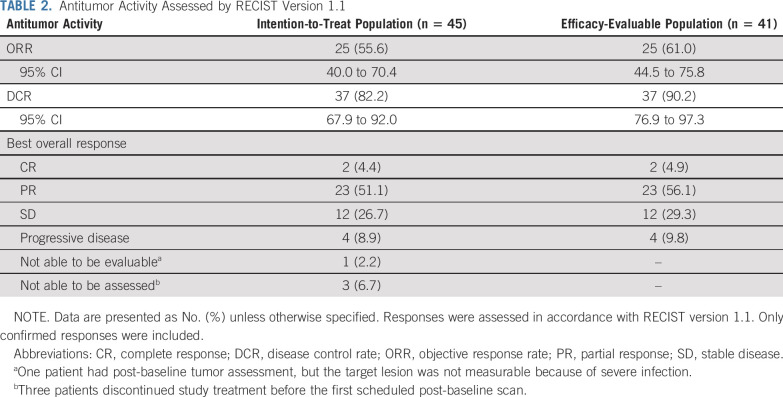
Antitumor Activity Assessed by RECIST Version 1.1

As of data cutoff, 22 patients (48.9%) had disease progression or had died. The median PFS was 8.8 months (95% CI, 5.6 months to not estimable; Fig 3B), and the 6-month PFS rate was 57.0% (95% CI, 40.2% to 70.7%). Eighteen deaths occurred (40%). The median OS was not reached (95% CI, 11.6 months to not estimable; Fig 3C), with a 9-month OS rate of 69.2% (95% CI, 52.9% to 80.8%).

### Safety

Forty-three patients (95.6%) in the safety population experienced at least one treatment-related AE (Table 3). Treatment-related grade 3 or 4 AEs occurred in 32 patients (71.1%), the most common of which were hypertension (24.4%), anemia (20.0%), and fatigue (15.6%). No treatment-related deaths occurred. Serious treatment-related AEs were observed in four patients (8.9%; Data Supplement), including grade 2 rash, grade 3 rash, and grade 4 pneumonitis (one patient [2.2%] for each AE); and grade 3 neutropenia, grade 3 anemia, and grade 4 thrombocytopenia (same patient).

**TABLE 3.**
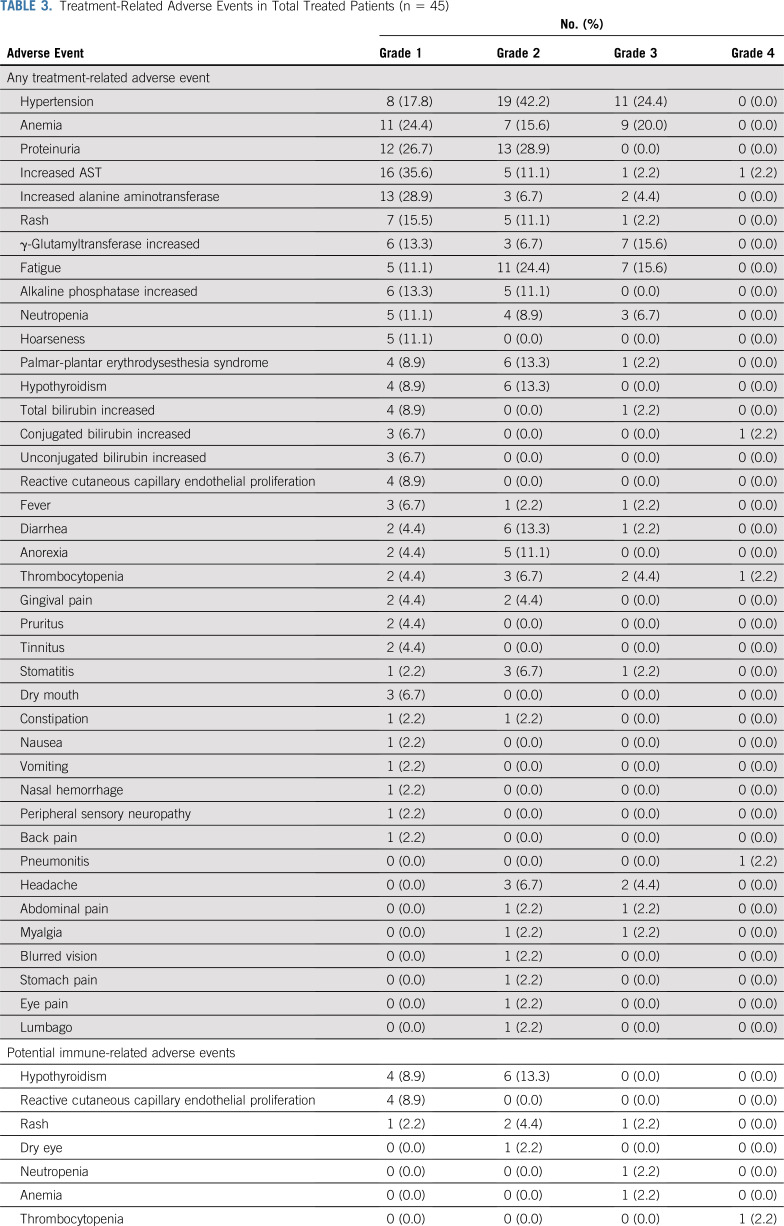
Treatment-Related Adverse Events in Total Treated Patients (n = 45)

Of 45 patients, 44 (97.8%) received at least one complete cycle of apatinib, whereas one discontinued treatment because of apatinib-related fatigue, stomatitis, and myalgia before completing the first cycle of apatinib. Forty-two patients (93.3%) required one or more dose interruptions for apatinib. Apatinib dose reductions were required by 33 patients (73.3%), of whom four (12.1%) required one dose reduction and 29 (87.9%) required two dose reductions. Five patients discontinued apatinib because of apatinib-related toxicities; of these, three continued the study with camrelizumab monotherapy, and two withdrew from the study permanently. The proportion of patients at each apatinib dose level and the reasons for apatinib dose reductions are summarized in the Data Supplement.

Fifteen patients (33.3%) had potentially immune-related AEs associated with camrelizumab. The most common potentially immune-related AE was hypothyroidism (22.2%; Table 3). Two patients had a total of four grade 3 or 4 potentially immune-related AEs (Data Supplement), one patient with grade 3 rash and another with grade 3 neutropenia, grade 3 anemia, and grade 4 thrombocytopenia. The patient with grade 3 rash had complete resolution with corticosteroids and resumed camrelizumab treatment. However, the patient with persistent grade 4 thrombocytopenia discontinued the study because treatment was delayed beyond 12 weeks, and tumor assessment at the time of discontinuation revealed disease progression. Four patients required corticosteroids: two with grade 2 rash; one with grade 3 rash; and one with grade 3 neutropenia, grade 3 anemia, and grade 4 thrombocytopenia.

### Post Hoc Analysis

**FIG 4.**
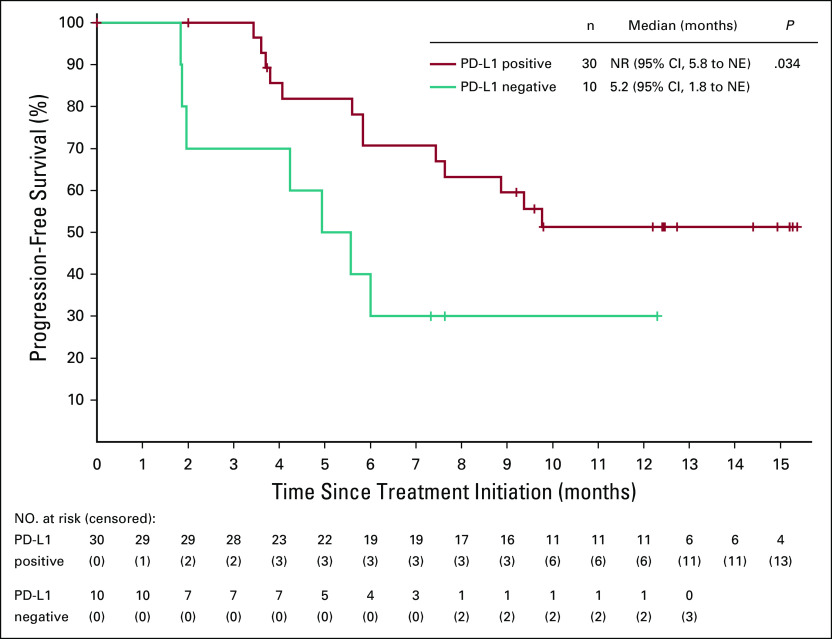
Kaplan-Meier estimates of progression-free survival by programmed death-ligand 1 (PD-L1) expression. Progression-free survival was assessed in patients with available PD-L1 expression (n = 40). We compared curves from the PD-L1–positive and the PD-L1–negative population using the log-rank test. NE, not estimable; NR, not reached.

No difference in ORR was observed between patients with PD-L1–positive and PD-L1–negative tumors (69.0% *v* 50.0%, *P* = .281; Data Supplement). However, patients with PD-L1–positive tumors had longer PFS than did those with PD-L1–negative tumors (Fig 4). The ORR was 77.8% (95% CI, 57.7% to 91.4%) in patients with SCC and 28.6% (95% CI, 8.4% to 58.1%) in patients with adenocarcinoma (Data Supplement). Patients with SCC had a prolonged PFS compared with those with adenocarcinoma (Data Supplement). No association was noted between response and p16 expression (Data Supplement).

## DISCUSSION

To our knowledge, this is the first reported study that evaluated the combination therapy of an anti–PD-1 antibody and a VEGFR inhibitor in patients with advanced cervical cancer. Our results revealed that camrelizumab plus apatinib showed promising antitumor activity, with a favorable response rate and durable response and a manageable toxicity profile in previously treated and advanced cervical cancer.

Patients with advanced cervical cancer who progress after first-line chemotherapy have few treatment options. Immune checkpoint inhibitors demonstrated antitumor efficacy in this setting, despite the modest response rates of 14.6% to 26%. Previous retrospective studies reported a response rate of 14.6% to 15.4% for apatinib monotherapy for patients with advanced cervical cancer with all histologic subtypes^18,19^ and 48% for patients with SCC.^17^ Our combination therapy achieved an ORR of 55.6% for all histologic subtypes of cervical cancer and 77.8% for patients with SCC (Data Supplement). These findings suggest that there is a combination effect of camrelizumab plus apatinib. It was reported that atezolizumab plus bevacizumab achieved no confirmed response in advanced cervical cancer, with a median PFS of 2.9 months, which led to termination of the trial at the first stage.^22^ However, cross-trial comparisons are difficult. Because bevacizumab is not approved for cervical cancer treatment by the China Food and Drug Administration, the proportion of patients treated with previous bevacizumab in our study was lower than in previous reports,^14,22^ and this might increase the response to apatinib. Nevertheless, evidence showed that patients might still respond to other VEGFR inhibitors after failure of first-line VEGF therapy.^23^ Whether the promising outcomes in this study were associated with different checkpoint and VEGF pathway inhibitors or patient selection remains to be investigated. The efficacy of apatinib monotherapy in advanced cervical cancer, as compared with in combination with camrelizumab, must be studied.

The safety profile of camrelizumab and apatinib was consistent with that reported for other anti–PD-1/PD-L1 antibodies and VEGF pathway inhibitors. Most AEs in our study, associated mainly with apatinib, were manageable. Hypertension was the most frequent AE attributed to apatinib, with a 84.4% incidence similar to that in the lenvatinib plus pembrolizumab trial in patients with endometrial cancer.^24^ Hypothyroidism, the most common immune-related AE, occurred in 22% of the patients, which was in line with that reported for camrelizumab^20,21^ and other PD-1 inhibitors.^14,25^ Some potentially overlapping toxicities of camrelizumab and apatinib should be noted (eg, hepatic toxicities, fatigue, and diarrhea). In our study, hepatic toxicities and diarrhea resolved in all patients by apatinib dose interruption and reduction (data not shown). The data suggest that these AEs were more likely the toxicities caused by apatinib rather than camrelizumab. Our findings were consistent with those of the pembrolizumab plus axitinib trial in renal cell cancer.^25^ However, the combinations of PD-1/PD-L1 and VEGF pathway inhibitors did report unacceptable toxicities in some studies.^26^

By far, the optimal dose of oral VEGFR inhibitors in combination therapy with PD-1/PD-L1 inhibitors remains unknown. A dose escalation study demonstrated that apatinib 250 mg once per day was the maximum tolerated recommended phase II dose when combined with camrelizumab 200 mg every 2 weeks.^21^ In a phase II trial for advanced triple-negative breast cancer, patients who had camrelizumab 200 mg every 2 weeks plus a higher starting dose of apatinib (250 mg day 1 to day 14) achieved a better response than did those with a lower starting dose (apatinib 250 mg day 1 to day 7).^27^ In our study, the proportion of apatinib dose reductions (73.3%) was high in contrast to that of a previous trial of lenvatinib plus pembrolizumab in endometrial cancer, which reported a 62.9% lenvatinib dose reduction.^24^ Only 35.6% of the patients tolerated the starting dose of 250 mg apatinib once per day in the third cycle of treatment (Data Supplement). Thus, a lower starting dose of apatinib may be appropriate for this combination in future trials.

Preclinical data showed that the normalization process produced by VEGF pathway inhibitors occurred in a dose-dependent manner.^16^ High doses of antiangiogenic agents resulted in a short vessel normalization window.^28^ In contrast, low doses of antiangiogenic agents may prolong vessel normalization and thereby reduce tumor hypoxia and enhance the infiltration of immune cells into tumors.^16^ In the current study, 19 of 23 patients who received more than six cycles of treatment received one half of the starting dose of apatinib (Data Supplement). However, whether the lower dose of apatinib leads to a durable response is unknown, and the optimal dose and schedule of such combinations must be further investigated.

In the KEYNOTE-158 trial, no responses were observed in patients with PD-L1–negative tumors.^14^ In our study, responses were observed in patients regardless of PD-L1 expression, although patients with PD-L1–positive tumors had prolonged PFS. Nevertheless, we acknowledge that the patient population was small, and this exploratory analysis was underpowered to distinguish responses between PD-L1–positive and PD-L1–negative tumors. The post hoc analysis revealed antitumor activity regardless of histologic subtype, despite a significant higher ORR and longer PFS among patients with SCC than among those with adenocarcinoma. PD-L1 expression is more frequent in SCC than adenocarcinoma in cervical cancer (54% *v* 14%) and non–small-cell lung cancer (52% *v* 17%).^29,30^ Given that higher PD-L1 expression has been associated with better outcomes of anti–PD-1/PD-L1 treatment, we suspected that the higher PD-L1 expression in SCC may to some extent contribute to the better response in this subgroup. However, no difference in PD-L1 expression was observed between SCC and adenocarcinoma in our study (*P* = .251, Data Supplement), which might be because of their limited sample sizes for comparison. Other features, including tumor mutational burden and tumor-infiltrating lymphocyte, may also contribute to different responses in patients with the two subtypes; this awaits additional investigations.

We acknowledge that this study has some limitations. First, this was a single-arm study with no control group for comparison, and thus selection bias could not be ruled out. Second, the small sample size reduces the certainty of effectiveness observed. Moreover, it was underpowered to compare across subgroups of patients with various PD-L1 expression and histologic subtypes.

Our data showed that camrelizumab combined with apatinib had promising antitumor activity and manageable toxicities in patients with advanced cervical cancer. Larger randomized controlled trials are warranted to validate our findings.

## Data Availability

The raw data that support the findings of this study are available from Sun Yat-sen University Cancer Centre (No. RDDA2020001554) but restrictions apply to the availability of these data, which were used under license for the current study, and so are not publicly available. Data are, however, available from the authors upon reasonable request and with the permission of Sun Yat-sen University Cancer Centre.
